# The Influence of Growth Mindset on the Mental Health and Life Events of College Students

**DOI:** 10.3389/fpsyg.2022.821206

**Published:** 2022-04-14

**Authors:** Weidong Tao, Dongchi Zhao, Huilan Yue, Isabel Horton, Xiuju Tian, Zhen Xu, Hong-Jin Sun

**Affiliations:** ^1^Department of Psychology, School of Teacher Education, Huzhou University, Huzhou, China; ^2^Department of Psychology, Neuroscience & Behaviour, McMaster University, Hamilton, ON, Canada

**Keywords:** growth mindset, life events, mental health, fixed mindset, college students

## Abstract

Growth mindset refers to our core belief that our talents can be developed through practice, which may influence our thoughts and behaviors. Growth mindset has been studied in a variety of fields, including education, sports, and management. However, few studies have explored whether differences in individuals’ growth mindsets influence college students’ self-reported mental health. Using the Growth Mindset Scale, Adolescent Self-rating Life Events Checklist, and SCL-90 Scale, data was collected from 2,505 freshmen in a University in China. Findings revealed that the students within the growth mindset group scored significantly lower on “mental health issues” and “stress due to life events” than the students in the fixed mindset group. Our findings suggest that individuals with a growth mindset are less prone to mental health problems than individuals with a fixed mindset.

## Introduction

### Current Status of Mental Health

Approximately 13.4% of adolescents suffer from mental health issues worldwide (Polanczyk et al., 2015). Findings from a nationwide epidemiological study in China found that the prevalence of depression and anxiety among college students was 11.7% (Chen et al., 2013), and 16.3% (Wu et al., 2015) respectively. Mood disorders are also common in China (Hardeveld et al., 2013; Gitlin and Miklowitz, 2016). The prevalence of mental disorders in China has continuously risen over the past 30 years (Huang et al., 2019). According to the statistics reported by the World Health Organization, the total number of people afflicted with depression worldwide is approximately 350 million, and the percentage of people globally suffering from depression is estimated at 4.4% (Smith, 2014; Liu et al., 2021). These statistics are also inclusive of children, with depression estimated to be the largest contributor of global burden of adolescent disease worldwide (Zheng and Zheng, 2015; Saxena and Kline, 2021). Poor interpersonal skills, poor grades, and lower graduation rates have been linked to mental health issues and suicidal behavior in college students (Tang et al., 2018).

Gender variables may also play a large role in mental health and wellbeing. A recent study demonstrated that gender was a major contributing factor to one’s self-rated health and functional wellbeing. Gender differences have also been demonstrated in physical and psychological functioning discrepancies along with many other health-related variables (Lorant et al., 2003; Gyasi and Phillips, 2018). Additionally, one’s socioeconomic status (SES) can have a large impact on mental health (Wagstaff and Doorslaer, 2000). The role of SES in the differential prevalence of depression is strongly supported by a large body of empirical evidence. That is, individuals who perceive themselves as having a low SES, are more susceptible to experiencing depressive symptoms (Sánchez-Moreno and Gallardo-Peralta, 2021; MacCarthy et al., 2022).

### Growth Mindset

The belief that one has the capacity to grow is known as a growth mindset. Growth mindset, or attributes that are malleable, encourage healthy and adaptive ways of facing and tolerating anxiety, frustration, and disappointment, which promotes resilience (Schroder, 2020). Growth mindset may make individuals more resilient and persistent in the face of challenges or difficulties, as they are more likely to adopt effort-oriented strategies in their efforts to achieve their goals (Zhao et al., 2021a). People with a growth mindset may suffer fewer stress and self-reported symptoms of psychological disease. For instance, when adolescents experience family stress, growth mindset can relieve the stress and reduce the protective effects of externalizing behaviors (Walker and Jiang, 2022). On the other hand, fixed mindset (entity theories of personality) have been shown to predict greater self-reported stress (Yeager et al., 2014) and anxiety following ostracism, as well as greater reports of psychosocial stress and psychopathology (Schleider et al., 2015; Miu and Yeager, 2016) compared to a growth mindset (incremental theories of personality; Yeager et al., 2016a). Entity theories of personality are associated with negative self-conscious emotions, such as shame (Yeager et al., 2011). Furthermore, findings from a meta-analysis study demonstrated a negative correlation between growth mindset and psychological distress (*r* = −0.220), a positive correlation between growth mindset and treatment value (*r* = 0.137), and a positive correlation between growth mindset and positive coping (*r* = 0.207; Burnette et al., 2020). While the impact of these findings were small, they support the notion that growth mindset was associated with psychological distress, treatment value, and positive coping.

Growth mindset refers to our core belief that our talents can be developed through practice, which may subsequently influence our thoughts and behaviors (Dweck, 2006). Mindset can affect one’s motivation, which in turn can affect academic resilience and performance. Both grit (Duckworth et al., 2007; Strayhorn, 2014) and mindset (Claro et al., 2016; Mccutchen et al., 2016; Wang et al., 2020; Sun et al., 2021) are linked to academic achievement, subjective wellbeing of the individual and the reduction of psychopathology (Clark and Malecki, 2022). A substantial body of research has shown that the belief that one’s abilities and talents can be developed (growth mindset), rather than fixed, can stimulate long-term learning. In terms of behavioral outcomes, growth mindset stimulates persistence in the face of obstacles and challenge-seeking behaviors and positively influences academic performance in primary, secondary, and higher education (Burnette et al., 2013; Jia et al., 2021). Specifically, growth mindset is significantly and positively correlated with academic performance (Wang et al., 2020) and predicts higher academic achievement than fixed mindset (Grant and Dweck, 2003; Blackwell et al., 2007). Moreover, a range of motivational factors, such as growth mindset, has been proven to positively affect individual performance such as mental rotation performance (Moè, 2016). However, growth mindset and grit are not always conducive to academic achievement (Bazelais et al., 2019). It is important to be cautious when drawing conclusions about the impact of growth mindset. But the effectiveness of growth mindset on academic performance cannot be ignored.

Additionally, growth mindset describes a type of mindset an individual may possess that describes their underlying beliefs about their ability to learn and their own intelligence (Dweck, 2006). For instance, if a student who has a growth mindset receives a poor grade on a test, they would likely put additional effort into studying for the next test, due to their core beliefs that they are able to grow, learn, and perform better. Thus, children with a growth mindset believe that they can develop their abilities through hard work, good strategies, and guidance from others (Blackwell et al., 2007; Haimovitz and Dweck, 2017). Having a growth mindset may also benefit individuals in a variety of different careers and educational fields, as it will allow them to persevere in the face of rejection and/or failure. In the 1980s, Dweck et al. developed a theory of implicit intelligence. They suggested that individuals would choose different achievement goals based on their own core beliefs, which would subsequently result in corresponding psychological and behavioral tendencies. These core beliefs were described in terms of having a growth mindset or a fixed mindset. That is, where individuals with a growth mindset will embrace challenges, constructive criticism, and will persevere in the face of setbacks, and individual with a fixed mind set will exhibit the opposite. Individuals with fixed mindset are more afraid of failure and mistakes. Thus, they will choose to avoid challenges which may result in failure, and will mistakenly attribute the failure to their incompetence, believing that only those who are not smart need to work hard.

### Benefits of Growth Mindset

Earlier studies in elementary and secondary schools have shown that a growth mindset is linked to better academic outcomes and lower levels of stress (Dweck, 2000; Rattan et al., 2012; Dweck and Yeager, 2019). Having a growth mindset has been demonstrated to be positively correlated with learning engagement and negatively correlated with perceived COVID-19 event intensity and stress (Zhao et al., 2021b). Furthermore, a positive correlation exists between the mindset of teachers and the appraisal of achievement for students who demonstrate increasing marks (De Kraker-Pauw et al., 2017). Moreover, students from lower-income families are less likely to maintain a growth mindset compared to their wealthier peers. However, the students who were able to maintain a growth-mindset were much more successful in overcoming the adverse effects of poverty on their academic achievement, then those who exhibited a fixed-mindset (Claro et al., 2016). The link between growth-mindset and academic performance may be explained by students’ behaviors such as learning strategies and learning habits (Lewis et al., 2020). In a study investigating the effects of growth mindset on navigation ability, a unique portion of variance in self-reported navigation ability was found, suggesting that a growth mindset can encourage people to seek out navigation challenges and train themselves to be better navigators in daily life (He and Hegarty, 2020). Additionally, research on the relationship between growth mindset and obesity in adolescents has demonstrated that those with a growth mindset are more likely to be successful at losing weight (Orvidas et al., 2018).

A positive correlation has been found between growth mindset and self-efficacy for health behaviors (Orvidas et al., 2018) and perceived control (Doron et al., 2009), such that individuals who possess a growth mindset are more likely to take control of their own health. Cognitive neuroscience approaches have been used to explore the mechanisms involved in mindset patterns, errors, and adjustments (Puusepp, 2021). Resilience has also been found to be related to improved attention to errors; that is, individuals with a growth mindset find it easier to bounce back from failures than individuals with a fixed mindset (Schroder et al., 2017b). Further, subtle feedback and information related to growth mindset could have a significant positive effect on students’ attitudes and motivation.

### The SCL-90 and ASLEC Applications

The Symptom Checklist-90-Revised (SCL-90) and Adolescent Self-rating Life Events Checklist (ASLEC) are self-report tools commonly used to measure clinical psychiatric symptoms and mental health status (Derogatis, 1983). The SCL-90 has been widely used to screen for mental health issues and is commonly used to assess the subjective mental health symptoms of college students (Xie and Dai, 2006). According to Ren (2009), the SCL-90 was used in 63.8% of the published articles regarding the mental health of college students, illustrating its strong validity and reliability. In addition, the Adolescent Self-rating Life Events Checklist (ASLEC) is a widely used self-assessment tool for measuring varying levels of life stress (Zhao et al., 2017).

Up to now, few studies have explored the relationship between growth mindsets, mental health and life events. The purpose of the current study is to explore whether individuals with a growth mindset might have better mental health and life event outcomes than those with a fixed mindset. We hypothesized that: (1) individuals with a growth mindset have better mental health than those with a fixed mindset; (2) individuals with a growth mindset have better self-report life events than those with a fixed mindset; and (3) individual family socioeconomic status will have an effect on growth mindset.

## Materials and Methods

### Participants

A total of 2,505 participants were recruited from Huzhou University in China. All participants were 1st-year college students (690 men, 27.54%; 1,815 women, 72.46%; mean age: 18.38 years; see Table 1). Informed consent was obtained from all participants. The study was reviewed and approved by the Institutional Review Board of the Human Research Ethics Committee of Huzhou University.

**Table 1:** Descriptive characteristics of participants.

	*N* or Mean (% or SD)
Gender	
Male	690 (27.54)
Female	1815 (72.46)
Self-report family economic	
Poor	219 (8.74)
General	1891 (75.49)
Relatively wealthy and wealthy	395 (15.77)
Birth place	
Countryside	830 (33.13)
Township	442 (17.64)
Non-provincial capital city	844 (33.69)
Capital city	389 (15.54)

### Measures

Data were collected in a computer room *via* online questionnaires that were divided into two sections. The first part consisted of a survey to gather demographic information of each participant, including gender, age, occupation, self-reported economic level, and place of birth. The second part included three scales: Growth Mindset Scale, SCL-90 Scale, and Adolescents Self-Rating Life Events Checklist (ASLEC).

The Growth Mindset Scale was created by Dweck (2006) and revised by Zhai (2018) in the Chinese version. The scale was used to measure the degree of growth mindset of participants. The Growth Mindset Scale is made up of six items and measures two dimensions: fixed mindset and growth mindset. A 6-point Likert scale was used to collect responses (1 indicated “disagree,” 6 indicated “strongly agree,” and 2, 3, 4, and 5 indicated varying degrees of agreement). The higher the score, the stronger the orientation of the dimension. Cronbach’s alpha for the six items in this study was 0.93.

The Symptom Checklist-90-Revised (SCL-90) was developed by Derogatis (1983) and revised by Tang (1999) in the Chinese version. SCL-90 has been a widely used self-report assessment of mental health (Hardt et al., 2012). It measures the degree of mental health on 10 dimensions, including somatization, obsessive–compulsive, interpersonal sensitivity, depression, anxiety, hostility, phobic anxiety, paranoid ideation, psychoticism, and other factors. The scale has 90 items, and which are scored from 0 to 4. Each question is rated according to how much the individual was bothered by the item in the last week on a five-point Likert scale (0 = “not at all,” 1 = “a little bit,” 2 = “moderately,” 3 = “quite a bit,” and 4 = “extremely”). Cronbach’s alpha for the SCL-90 was 0.96 in the present study.

The Adolescents’ Self-Rating Life Events Checklist (ASLEC; Liu et al., 1997; Hardt et al., 2012) was used to assess the frequency and intensity of stressful life events among adolescents, especially middle school students and college students. The scale included six factors, including interpersonal relationships, study pressure, being punished, bereavement, pressure of health and adaptation, and other factors. Within the six factors there are 27 items, which are scored from 1 to 5 (1 = “occurred but exercised no influence” to 5 = “occurred and exercised a severe influence”), with higher summed scores indicating higher levels of life stress. Each subscale has a total score based on subscale entries. In the present study, the Cronbach’s alpha for the ASLEC was 0.85. The scale is generally used to measure the stress levels of children and adolescents with negative life events (Wang et al., 2016).

### Statistical Analyses

Statistical analyses were performed using the SPSS 22.0. Descriptive analyses were used to examine the descriptive characteristics of the study population. We used the independent samples T-test to test gender differences in the results on the growth mindset. A one-way ANOVA was used to test the degree of socioeconomic status (SES) factor and the place of birth factor. The significance level was set at *p* < 0.05.

## Results

### Differences in the Results of Growth Mindset Scale Due to Gender and SES

There was no significant difference between men and women [*t* (2503) = 1.53, *p* > 0.05] in having a growth-mindset. The degree of SES was divided into four levels. Since the wealthy sample size was too small [*N* = 18], SES was further divided into three levels: poverty, ordinary, and preferably. A significant difference was found between the varying SES groups [*F* (2, 2,502) = 6.30, *p* < 0.01]. Birth of place factors were divided into four categories: rural area, township, non-provincial capital cities, and provincial capital cities. Result showed that there was no significant effect of the place of birth factor on growth-mindset [*F*(3, 2,501) = 2.37, *p* > 0.05].

### One-Way ANOVA of Adolescents Self-Rating Life Events Checklist and SCL-90 Scale

**Figure 1:**
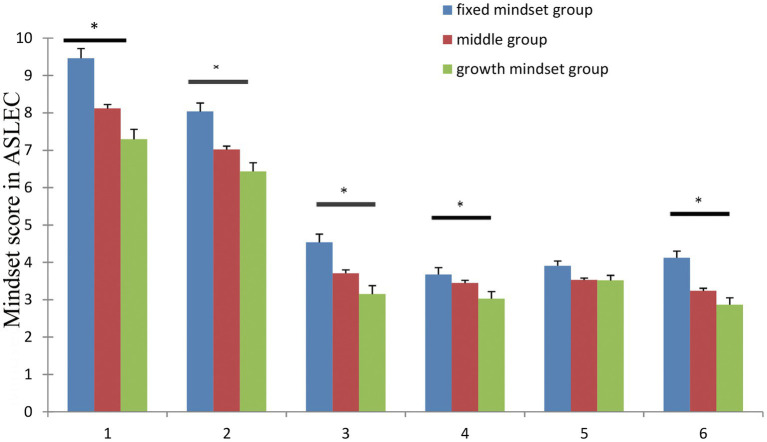
Mean score of subscale of Adolescent Self-rating Life Events Checklist in different groups with error bars representing standard error of the mean and asterisks representing significant difference among groups.

Based on individual growth-mindset scores, participants were divided into three groups: top growth mindset group with 27% having scores between 26 and 36 (687 participants), middle group with scores between 19 and 25 (1,189 participants), and the fixed mindset group with scores between 6 and 18 (629 participants). A main effects of group on study pressure [*F* (2, 2,502) = 12.02, *p* < 0.001], interpersonal relationships [*F* (2, 2,502) = 20.93, *p* < 0.001], being punished [*F* (2, 2,502) = 8.81, *p* < 0.01], bereavement [*F* (2, 2,502) = 4.95, *p* < 0.01], and others [*F* (2, 2,502) = 14.45, *p* < 0.001] was seen (Figure 1; Table 2). However, no significant differences were seen for pressure of health and adaptation [*F* (2, 2,502) = 2.72, *p* > 0.05].

**Table 2:** One-way ANOVA of Adolescents Self-Rating Life Events Checklist.

	M (SD)	*F*	*P*
1. Interpersonal relationship	8.19 (4.64)	20.93	< 0.001
2. Study pressure	7.08 (4.05)	12.02	< 0.001
3. Being punished	3.74 (3.96)	8.81	< 0.01
4. Bereavement	3.42 (3.25)	4.95	< 0.01
5. Pressure of health and adaptation	3.57 (2.36)	2.73	> 0.05
6. Others	3.30 (3.16)	14.56	< 0.001

**Figure 2:**
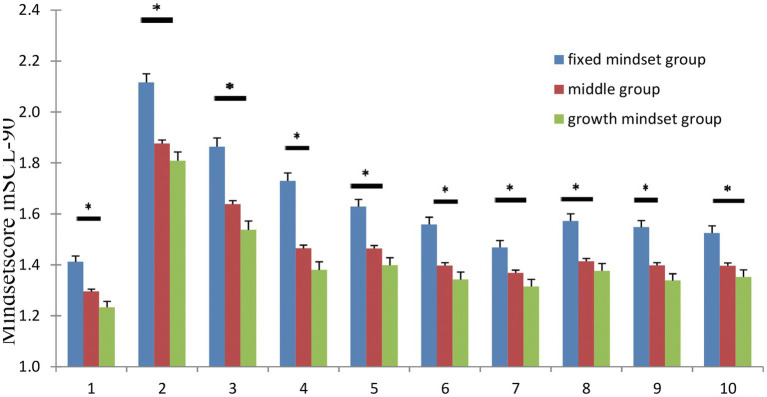
Mean score of subscale of SCL-90 in different groups with error bars representing standard error of the mean and asterisks representing significant difference among groups.

A one-way ANOVA was conducted on the SCL-90 scale scores. Results showed there was a significant main effect of group on somatization [*F* (2, 2,502) = 12.21, *p* < 0.001], obsessive–compulsive [*F* (2, 2,502) = 24.13, *p* < 0.001], interpersonal sensitivity [*F* (2, 2,502) = 23.95, *p* < 0.001], depression [*F* (2, 2,502) = 30.41, *p* < 0.001], anxiety [*F* (2, 2,502) = 13.03, *p* < 0.001], hostility [*F* (2, 2,502) = 15.35, *p* < 0.001], phobic anxiety [*F* (2, 2,502) = 6.83, *p* < 0.01], paranoid ideation [*F* (2, 2,502) = 8.13, *p* < 0.001], psychoticism [*F* (2, 2,502) = 11.94, *p* < 0.001], and other [*F* (2, 2,502) = 9.96, *p* < 0.001; Figure 2; Table 3].

**Table 3:** One-way ANOVA of SCL-90 Scale.

	M (SD)	*F*	*p*
1. Somatization	1.30 (0.41)	12.21	< 0.001
2. Obsessive–compulsive	1.90 (0.61)	24.14	< 0.001
3. Interpersonal sensitivity	1.65 (0.61)	23.95	< 0.001
4. Depression	1.49 (0.56)	30.41	< 0.001
5. Anxiety	1.48 (0.51)	13.03	< 0.001
6. Hostility	1.41 (0.51)	15.35	< 0.001
7. Phobic anxiety	1.37 (0.48)	6.83	< 0.01
8. Paranoid ideation	1.43 (0.50)	8.18	< 0.001
9. Psychoticism	1.41 (0.46)	11.94	< 0.001
10. Other	1.41 (0.49)	9.96	< 0.001

### Correlation Analysis

A significant negative correlation was found between growth mindset and SCL-90 [*r* = −0.145, *p* < 0.001]. A significant negative correlation was found between growth mindset and ALSF (*r* = −0.13, *p* < 0.001), and a significant positive correlation between life event and mental health (*r* = 0.563, *p* < 0.001).

## Discussion

The current study aimed to investigate whether growth mindset has an influence on an individual’s mental health status and life events compared to having a fixed mindset. Two main findings came out from the present study. First, SES has a significant effect on the likelihood of having a growth mindset, such that, the higher one’s SES, the less likely that they will have a growth mindset. Second, the growth mindset group scored significantly lower on SCL-90 and ALSEC than the fixed mindset group. These results suggest that that having a growth mindset has a positive effect on one’s mental health.

### Gender Difference and SES in Growth Mindset

We found no significant difference between gender and mindset, suggesting that gender does not play a contributing role in determining whether one will have a growth or fixed mindset. These results are consistent with previous studies (Heyman et al., 2002; Kornilova et al., 2009; Yan et al., 2014; Tucker-Drob et al., 2016; Macnamara and Rupani, 2017). The differences in self-reported SES had a significant effect on growth mindset, such that individuals of lower SES were found to have greater growth mindset. Findings regarding the effect of SES and growth mindset have been mixed in the literature. Some studies have demonstrated that those with higher SES had higher growth mindset, while those with lower SES had lower growth mindset (Claro et al., 2016; Yeager et al., 2016b; Miyamoto et al., 2018; Destin et al., 2019; Bernardo, 2021). The reason behind our findings may be due to the face that compared to individuals of higher SES, poorer students hold a stronger belief that they can bridge the gap by working hard and persevering (Burnette et al., 2017). That is, individuals who are raised in families of high SES were more likely to have been often praised and rewarded for their work. This in turn, may have resulted in greater fear of failure and challenge later in life, resulting in a more fixed mindset than in their lower SES counterparts.

There were no significant differences in the place of birth of students. These findings likely reflect the degree of the gap of socioeconomic status, as the gap between the rich and the poor may be narrowing due to the economic development. Another possible explanation rests in our participant sample. The sample of this study comes from an undergraduate university. As an undergraduate who come from low SES families need to make much more efforts to succeed in China. Therefore, they tend to form growth mindset.

In regard to mental health status and growth mindset, our findings demonstrated that college students with a growth-mindset had lower scores on the SCL-90 and ASLEC scales, while students with a fixed mindset had higher scores on these two scales, indicating that individuals with a growth mindset have greater mental health and better life event perception ability. As a result, having a growth mindset likely provides an individual with potential life benefits. In the present study, the association between the number of stressful life events and post-traumatic stress symptoms, depression, substance use, and non-suicidal self-injury was lower in the growth mindset group than in the fixed mindset group. These results suggest that having a growth mindset increase one’s resilience to poor mental health (Schroder et al., 2017a).

### The Practical Implications of Growth Mindset

Our findings also demonstrated that individuals who had the greatest growth mindset were healthier on most subscales of the SCL-90 and ASLEC scales. This may be due to the fact that the growth mindset group underestimated their self-assessment when they filled out self-report questionnaires. Mindsets are conceptualized as a framework through which individuals can explain their domain-related experiences that influence subsequent thoughts, emotions, goals, and behaviors (Dweck et al., 1995). Students with a growth mindset interpret mistakes as learning opportunities, stay focused when facing challenges, and tend to underestimate their own symptoms on the SCL-90 and ASLEC. They are more cautious and focused when completing the scales, resulting in lower scores. In contrast, students with fixed mindsets view mistakes as a sign of lack of competence and tended to escape when faced with challenges. They are more likely to overestimate their own symptoms and pay attention to the results when completing the scale.

The current study also found that groups with different mindsets might have different abilities in terms of life event perception. Individuals with a growth mindset demonstrated better life perception ability than those with a fixed mindset. In terms of study stress factors, individuals with a growth-mindset scored lower on the study pressure subscale. Individuals with a growth mindset may take initiative and derive strategies to adjust their thoughts, modify their expected goals in time, and maintain an optimistic and positive attitude. In contrast, individuals with a fixed mindset might adhere to goals when facing learning pressure, and if they fail to achieve their goals, demonstrate increased pressure and negative emotions. College students with a growth mindset show better adaptability when facing difficult situations or challenges, and appear to be more optimistic. They believe that their abilities can be cultivated and that they can achieve their goals through hard work and perseverance. However, college students with a fixed mindset believe that their abilities are stable, and that they cannot be improved through effort. When they have difficulties, they may avoid challenges and demonstrate negative coping mechanisms.

Another possible explanation for these findings is that the growth-mindset group may have more positive coping strategies, and therefore might have better mental health and lower life event awareness than the fixed mindset group. The study pressure factor in ASLEC included questions such as “unsatisfactory test results,” “heavy study burden,” “family financial difficulties,” “expected (good students) failure,” and “pressure to enter school.” Individuals with a fixed mindset might employ more negative coping strategies than those with a growth mindset. Students with a fixed mindset often have external motivation to learn or work (Dweck, 2006). They tend to demonstrate much more negative coping strategies for justifying failures, such as finding excuses for failures. In fact, this occupies the cognitive resources of learning and hinders their own development (Dweck, 2006; Dweck et al., 2014). However, students with a growth mindset demonstrate much more positive coping strategies. They analyze their own reasons and believe that hard work can improve their performance and ability. They mobilize all available cognitive resources to promote their own development. These individuals are more likely to overcome difficult academic transitions (Yeager et al., 2014), and to embrace active learning strategies, compared to students with a fixed mindset (Cavanagh et al., 2018).

### Future Research

The primary limitation of this study is a lack of investigation on the transformation of individuals with a fixed mindset to a growth mindset through intervention. Future studies should employ certain interventions that allow for this measurement. Furthermore, this study was unable to answer whether better mental health of growth mindset individuals is due to higher self-assessment or more positive coping strategies. Nonetheless, our findings support the results of previous research that college students with a growth mindset have better overall mental health than college students with a fixed mindset. Future studies could explore neural mechanisms underlying the possession of a growth mindset versus a fixed mindset. Furthermore, future studies ought to investigate the effects of growth mindset training on individuals with a fixed mindset.

## Data Availability Statement

The raw data supporting the conclusions of this article will be made available by the authors, without undue reservation.

## Ethics Statement

The study was reviewed and approved by the Institutional Review Board of the Human Research Ethics Committee of Huzhou University. Written informed consent to participate in this study was provided by the participants’ legal guardian/next of kin.

## Author Contributions

WT and HY: conceptualization. DZ and ZX: formal analysis. DZ: visualization. WT, DZ, and XT: writing—original draft. H-JS and IH: writing—review and editing. All authors contributed to the article and approved the submitted version.

## Funding

This work was supported by the Ministry of Education in China, Project of Humanities and Social Sciences (project no. 18YJA760052), and Zhejiang Educational Science Planning Project (project no. 2018SCG039).

## Conflict of Interest

The authors declare that the research was conducted in the absence of any commercial or financial relationships that could be construed as a potential conflict of interest.

## Publisher’s Note

All claims expressed in this article are solely those of the authors and do not necessarily represent those of their affiliated organizations, or those of the publisher, the editors and the reviewers. Any product that may be evaluated in this article, or claim that may be made by its manufacturer, is not guaranteed or endorsed by the publisher.
